# Correlation between Preoperative Ultrasonographic Findings and Clinical, Intraoperative, Cytopathological, and Histopathological Diagnosis of Acute Abdomen Syndrome in 50 Dogs and Cats

**DOI:** 10.3390/vetsci4030039

**Published:** 2017-08-08

**Authors:** Ahmed Abdellatif, Martin Kramer, Klaus Failing, Kerstin von Pückler

**Affiliations:** 1 Department of Veterinary Clinical Science, Clinic for Small Animals (Surgery), Justus-Liebig University (JLU), 35392 Gießen, Germany; Martin.Kramer@vetmed.uni-giessen.de (M.K.); Kerstin.H.Pueckler@vetmed.uni-giessen.de (K.v.P.); 2 Animal Surgery Department, Assiut University, Assiut 71515, Egypt; 3 Unit for Biomathematics and Data Processing, Veterinary Faculty, Justus-Liebig University (JLU), Gießen 35392, Germany; Klaus.Failing@vetmed.uni-giessen.de

**Keywords:** dog, cat, ultrasonography, acute abdomen, laparotomy

## Abstract

Acute abdomen syndrome is an emergency in small animal practice that requires rapid diagnosis to determine the appropriate treatment. No studies have correlated the preoperative abdominal ultrasonography (US) findings with the clinical, surgical, cytopathologic, and histopathologic findings. This retrospective study was designed to evaluate abdominal US in the diagnosis of acute abdomen syndrome using surgery as a “criterion standard”. The most frequently misinterpreted lesions with US were also identified. The study included 50 dogs and cats with physical examination, an US diagnosis, US guided fine-needle aspiration cytology, intraoperative findings, and histopathology. Intraoperatively, 49 primary and 43 secondary lesions were identified. The sensitivity, specificity, and positive and negative predictive values for US were calculated. There was a good agreement between the US diagnosis and intraoperative findings of 86.9% (80/92), for both primary and secondary lesions (*p* < 0.0001). Cytology and histopathology examinations corroborated the US in 86.4% (*n* = 64/74) of primary and 66.2% of secondary (*n* = 49/79) lesions. Using US as the “criterion standard”, the sensitivity of abdominal palpation for identification of ascites and masses was 32.4% and 43.7%, respectively, while the specificity was 93.7% and 94.4%, respectively. Abdominal US is a useful preoperative modality for diagnosing acute abdominal diseases in dogs and cats. Care should be taken with interpretation of gastrointestinal perforation, omental tumors, and common bile duct rupture, as these lesions are frequently misinterpreted with US.

## 1. Introduction

Acute abdomen syndrome is a clinical syndrome characterized by the sudden onset of severe abdominal pain accompanied by signs and symptoms of abdominal involvement [1,2,3,4]. Acute abdomen syndrome may be associated with a wide variety of disease processes, ranging from self-limiting conditions to surgical emergencies. Disorders of the gastrointestinal, hepatobiliary, or urogenital systems, the spleen, pancreas, and peritoneum have been reported [3,4,5]. Acute abdomen syndrome requires rapid, careful evaluation, and integration of clinical findings, clinical pathology, and diagnostic imaging to determine the appropriate medical and/or surgical management [4,6,7].

Abdominal ultrasonography (US) is a valuable procedure for the diagnosis of a wide range of acute abdomen diseases [1,2,3,4,6,8]. Ultrasound findings of a target-like mass of concentric hyperechoic and hypoechoic rings is diagnostic of intestinal intussusception in dogs and cats [9,10]. Transmural thickening with loss of normal gastrointestinal wall layering and evidence of local lymphadenopathy is indicative of canine and feline intestinal adenocarcinoma [11,12]. Ultrasonographic appearance of an enlarged and hypoechoic pancreas with ascites, hyperechoic peripancreatic tissues, and extrahepatic biliary obstruction is suggestive of pancreatitis [1,13]. In a study of 12 dogs with gallbladder and extrahepatic biliary tract obstruction, US correctly diagnosed obstruction in 10 cases. However, the cause of obstruction could not be determined by abdominal US alone in two cases [14]. In a series of 129 human patients with ovarian cancer, ultrasound had low sensitivity and specificity in patients with lesions less than 2 cm [15].

The purpose of this retrospective study was to evaluate the performance of US as a pre-surgical diagnostic tool in patients with acute abdomen syndrome, and to identify the most frequently misinterpreted lesions. In addition, the ability of abdominal palpation to identify the presence of ascites or masses was evaluated. We hypothesized that abdominal US is useful pre-surgically for the diagnosis of canine and feline acute abdomen disease.

The present study is similar to a previous study that described US features of acute abdomen [1] and a study comparing US and exploratory laparotomy in the dog and cat [16]. However, our study provides cytopathologic and histopathologic identification of lesions. Furthermore, our study compares abdominal palpation with US for the detection of peritoneal effusion and masses.

## 2. Materials and Methods

This retrospective study was performed at the Clinic for Small Animals Surgery of the Justus Liebig University in Giessen. Surgical and US records of dogs and cats admitted for acute abdomen syndrome were reviewed. Inclusion criteria were: animals with preoperative physical examination, US, US guided fine-needle aspiration cytology, and laparotomy. Clinical records included a complete history, breed, age (months/years), sex (male/female), weight, and duration of clinical signs (hours/days). The patient’s general condition was categorized to one of five clinical stages: undisturbed (1); slightly disturbed (2); moderately impaired (3); severely impaired (4); and anesthetized or comatose animals (5).

Abdominal palpation findings were categorized into one of four groups: normal (1); tense abdomen (2); suspicion of ascites (3); and palpable abdominal mass (4).

Records of thoracic and abdominal radiographs were reviewed for evidence of abnormalities involving the parenchymal organs, gastrointestinal tract (GIT), and peritoneal cavity. The US examinations were performed by one of three radiologists, each with more than four years of experience (board-certified radiologists, ECVDI). As a routine procedure in our clinic, the linear transducer (7.5 MHz to 14 MHz) is used to image cats and small dogs (<20 kg); while large dogs (>20 kg) are usually examined with a convex transducer providing greater tissue penetration (5 MHz), typically in combination with a high frequency linear probe (Toshiba, Imaging Systems GmbH, Neuss, Germany).

Each abdominal organ was examined for size, shape, echotexture, and echogenicity. Scanning was performed in dorsal or lateral recumbency. The examination began at the urinary bladder. A standardized template for US was used for all examinations and included any identified abnormalities. Abdominal structures were considered free of abnormalities if no abnormalities were documented in the written US report. To facilitate statistical analysis, the final intraoperative and US diagnoses were recorded in an excel file with a numeric system (1 = intestinal tumor, 2 = stomach tumor, 3 = liver tumor, 4 = splenic tumor, etc.). The numeric system included 20 different diagnoses and one to three diagnoses were listed for each patient. The intraoperative findings were classified according to the anatomical location and gross pathology. The intraoperative reports included the surgeon’s remarks and the techniques used. The intraoperative findings were classified as primary or secondary lesions, as designated by the surgeon, and according to the organs involved.

A primary lesion is the main intraoperative finding, while secondary lesions include other associated lesions. For instance, in an animal with a gastrointestinal foreign body, intestinal perforation, and septic peritonitis, the foreign body is the primary lesion, and intestinal perforation, and septic peritonitis the secondary lesions.

Preoperative US guided fine-needle aspiration cytology was performed in 50 patients and postoperative histopathology in 48. Two patients were humanely euthanized during surgery at the owner’s request and no histopathology was available. Results from cytology were classified into different categories: ascitic fluid, transudate (1); ascitic fluid, modified transudate (2); ascitic fluid, purulent exudates (3); and hemoperitoneum (4). Cytologic and histopathologic findings of mass lesions were classified into six categories: benign epithelial (1a); malignant epithelial (1b); benign mesenchymal (2a); malignant mesenchymal (2b); lymphatic malignancies (3); neuroendocrine malignancies (4); purulent inflammation (5); and inflammatory or hyperplastic (6).

Statistical analysis was done with the statistical program packages BMDP [17] and BiAS [18] for Windows. For data that met the assumption of normality, mean, minimum, maximum, and standard deviation (SD) were calculated. Fisher’s exact test was used to determine the association between preoperative US, intraoperative findings, and abdominal palpation. The sensitivity, specificity, and positive and negative predictive values for US were calculated using the surgical diagnosis as the “criterion standard”. To assess the value of abdominal palpation, the sensitivity and specificity were calculated using the US as the “criterion standard”. Additionally, the 95% confidence intervals (CI) were calculated. For all tests, a significance level of 0.05 was chosen and a value of *p* < 0.05 was considered statistically significant.

## 3. Results

Fifty animals met the inclusion criteria. There were 33 dogs (66%) and 17 cats (34%). Thirty-three animals were male (66%) and 17 were female (34%). The mean age of the dogs was 7 ± 4.6 years (range, 3 months to 15 years), and that of the cats was 5.7 ± 4.7 years (range, 7 months to 15 years). The mean weight of dogs was 19.7 ± 13.3 kg (range, 3–50 kg) and that of cats was 4.6 ± 1.5 kg (range, 2.7–7.9 kg). The mean duration of clinical signs prior to presentation was 2.9 ± 1.7 days in dogs (range, 2 h to 5 days), and 4.3 ± 3.7 days (range 2 h to 10 days) in cats. On the day of the first examination, 56% (*n* = 28) had clinical signs for less than three days. In most cases, nonspecific clinical signs including vomiting, abdominal pain, lethargy, anorexia, weight loss, and depression were reported. On physical examination, patients were undisturbed (*n* = 11), slightly disturbed (*n* = 12), moderately impaired (*n* = 12), severely impaired (*n* = 13), and anesthetized (*n* = 2).

**Figure 1:**
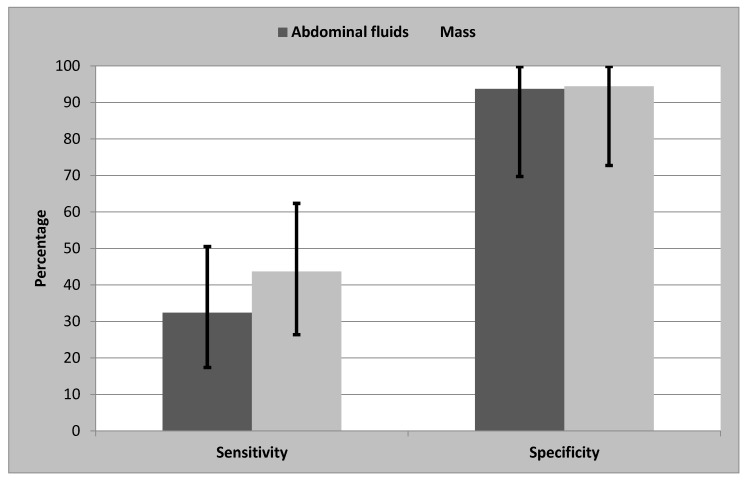
Bar graph showing the sensitivity and specificity of abdominal palpation for the detection of fluid and masses, when compared to ultrasonography. The black error bars denote the 95% confidence intervals.

Abdominal palpation identified acute abdominal disease in 76% of patients (*n* = 38) and identified an abdominal mass (*n* = 14), ascites (*n* = 11), and tense abdomen (*n* = 13). The abdomen was palpably normal in 10 animals. Ultrasound was used to confirm abdominal palpation of ascites or masses. Palpation of fluid had a sensitivity of 32.4% (CI: 17.4–50.5%) and specificity of 93.7% (CI: 69.7–99.8%) (Figure 1). Palpation of an abdominal mass had a sensitivity of 43.7% (CI: 26.4–62.3%) and a specificity of 94.4% (CI: 72.7–99.9%). There was a significant association between palpation and US diagnosis of ascites (*p* < 0.05), but no significant relationship between palpation and US detection of an abdominal mass (*p* > 0.05).

Ninety-two intraoperative lesions (49 primary and 43 secondary) were documented. The preoperative diagnosis in one case was an intestinal foreign body. However, at surgery, the abdomen was normal. Intraoperative findings are summarized by anatomical location in Table 1. Primary lesions were most frequently located in the gastrointestinal tract (53%, *n* = 26), the peritoneum and peritoneal cavity (14.3%, *n* = 7), and pancreas (12.2%, *n* = 6). Secondary lesions were most commonly located in the peritoneum and peritoneal cavity (60.5%, *n* = 26), gastrointestinal tract (14%, *n* = 6), and spleen (9.3%, *n* = 4).

Thoracic and abdominal radiography did not identify the primary lesion in 71% (35/49) of cases, while secondary lesions were not identified in 41.8% (18/43). Normal radiographic findings were reported in 36% (18/50), while loss of serosal detail was the most common abnormal finding in 32% (16/50). Gastrointestinal obstructions (*n* = 8), splenic tumors (*n* = 2), kidney tumors (*n* = 2), nonspecific intra-abdominal mass (*n* = 2), liver mass (*n* = 1), and intussusception (*n* = 1) were suspected by radiography.

Comparing preoperative abdominal US and intraoperative diagnoses, there was a significant agreement of 86.9% (80/92) for both primary and secondary lesions (*p* < 0.0001). The sensitivity, specificity, and positive and negative predictive values of US with confidence intervals are summarized in Table 2. Ultrasound findings were corroborated by cytology in 86.4% of the lesions (*n* = 64/74) and histopathology in 66.2% of the lesions (*n* = 49/74) (Table 3).

Surgical lesions were not identified not identified by US in 13% (12/92) of lesions. False negative diagnoses were reported in 10.2% (5/49) of primary lesions. This included GIT perforation (*n* = 2), ruptured common bile duct (*n* = 2), and intestinal foreign body (*n* = 1) (Table 4). False positive diagnoses were reported in 14% (7/50) of cases, and included omental tumors (*n* = 2), intestinal neoplasia (*n* = 2), pancreatic tumor (*n* = 1), pancreatic abscess (*n* = 1), and intestinal foreign body (*n* = 1). There was disagreement in the diagnosis of secondary lesions between the US and surgical findings in 16.2% of the secondary lesions (*n* = 7/43). This included omental tumors (*n* = 3), gastrointestinal tract perforation (*n* = 2), generalized peritonitis (*n* = 1), and an intestinal ulcer (*n* = 1). The primary lesions in these cases were correctly diagnosed preoperatively. A false positive diagnosis of the secondary lesions was reported in 9.3% (4/43), and included splenic tumors (*n* = 2), lymphadenopathy (*n* = 1), and pancreatic abscess (*n* = 1).

Lesions of the gastrointestinal tract were reported in 34.8% (32/92); 53% (26/49) were primary and 14% (6/43) were secondary. The pathological conditions included intussusception (*n* = 9), intestinal neoplasia (*n* = 7), foreign body (*n* = 5), perforation (*n* = 4), gastrointestinal ulcer (*n* = 4), and gastric tumor (*n* = 3). Intussusception was accurately diagnosed by US in all cases (9/9). Secondary lesions including omental masses were diagnosed in one case. Intussusceptions were confirmed histologically, with enteritis and lymphadenitis as the most reported findings. Intestinal neoplasia was suspected by US in nine cases and intestinal masses were identified intraoperatively in seven cases (7/9). Histopathology corroborated the US suspicion in five cases (5/9) of lymphoma (*n* = 3) and fibrosarcoma (*n* = 2), while purulent inflammation was documented in four cases. Four of five cases of gastrointestinal foreign body suspected with US were confirmed at surgery. In one case (1/0), a false positive diagnosis was reported by the US. In another case (*n* = 1), the false negative diagnosis was reported by the US. Gastrointestinal perforation location was not detected with abdominal ultrasound (0/4).

Gastrointestinal ulcer was suspected by US and corroborated by histopathology in three cases (3/4). The etiologies were: neoplasia (*n* = 3) and gastric foreign body. Gastric tumors were diagnosed accurately by US in all cases (3/3) and were identified as gastric carcinoma on histopathology. Ulcerative gastritis was reported on histopathology in the case of foreign bodies.

Diseases of the peritoneum and peritoneal cavity were detected in 35.9% (*n* = 33/92) of lesions and included primary (14.3%, 7/49) and secondary (60.5%, 26/43) lesions. Generalized peritonitis (*n* = 13), peritoneal effusion (*n* = 9), focal peritonitis (*n* = 6), and omental masses (*n* = 5) were identified.

Ultrasound correctly identified generalized peritonitis and peritoneal effusions in 12 of 13 cases. A history of previous abdominal surgery was reported in two animals. Purulent exudate was reported in all cases and purulent inflammation was diagnosed in the omentum, liver, and stomach. Ultrasound accurately diagnosed peritoneal effusion as a primary lesion (*n* = 1) and a secondary lesion (*n* = 8). Cytological examination indicated a modified transudate (*n* = 7) and purulent exudate (*n* = 2). Focal peritonitis as a secondary lesion was suspected by US and confirmed intraoperatively in six cases. Cytopathology was available in one case and reported chronic necrotizing peritonitis and omentitis.

Five omental masses were diagnosed. The masses measured approximately 1 cm (*n* = 3), 3 cm (*n* = 1), and 15 cm (*n* = 1). However, only the large (3 cm and 15 cm) omental masses were detected by US and were classified as lymphadenopathy and a splenic tumor (false positive diagnosis). The masses were identified as benign hyperplasia (3/5), omental fibrosarcoma (1/5), and lymphoma (1/5).

Pancreatic disease represented 7.6% (7/92) of all lesions including primary (12.2%, 6/49) and secondary (2.3%, 1/43) lesions. Pancreatic abscess (*n* = 5) and neoplasia (*n* = 2) were diagnosed. Ultrasound identified pancreatic abscess in seven cases (7/5), four of which were confirmed by histopathology (4/7). In three cases, the pancreas was normal. Pancreatic neoplasia was suspected by US in three cases (3/2) but was not corroborated by histopathology.

Hepatic lesions were identified in 7.6% (7/92) of lesions as primary (8.2%, 4/49) and secondary (7%, 3/43) lesions. Hepatic masses (*n* = 5) and ruptured common bile duct (*n* = 2) were diagnosed. Based on US, nodular hyperplasia (*n* = 3), neoplastic infiltration (*n* = 1), and abscessation (*n* = 1) were suspected. Fine needle aspiration and postoperative pathology diagnosed extra medullary hematopoiesis in three cases, while hepatic neoplasia and hepatic abscess were confirmed with cytology (2/2). The bile duct lesions were not diagnosed by US (0/2), but cholangitis, pericholecystic echogenic reaction, and duct obstruction were identified.

Splenic masses represented 5.4% (5/92) of lesions as primary (2%, 1/49) and secondary (9.3%, 4/43) lesions. With US, malignant splenic neoplasia was suspected in six cases and confirmed in four cases (4/6). A large mass in the omentum (1/6) and normal spleen (1/6) were reported in the other two cases. Extra-medullary hematopoiesis (*n* = 3), regenerating nodules and nodular hyperplasia (*n* = 2), and omental fibrosarcoma (*n* = 1) were diagnosed by histology. Splenic abscessation was diagnosed preoperatively in one case (1/5) and was confirmed with histopathology.

Lymph node disease was reported in 6.5% (6/92) as primary (6%, 3/49) and secondary (7%, 3/43) lesions. Ultrasound correctly identified all cases (6/6); in three cases, purulent lymphadenitis was the primary US differential diagnosis, followed by neoplasia; the opposite was reported in the other three cases. Histopathology confirmed the US diagnosis as purulent lymphadenitis (3/6), malignant lymphosarcoma (2/6), and undifferentiated blastoma (1/6). A false positive US diagnosis of lymphadenopathy (1/0) was diagnosed intraoperatively as an omental tumor and corroborated by histology as nodular hyperplasia of the omentum.

Kidney lesions were detected in 2.1% (2/92) as primary lesions (4%, 2/49). Ultrasound accurately diagnosed renal neoplasia (*n* = 1) and urinoma (*n* = 1); all were accurately diagnosed by abdominal ultrasound. With histological examination, a malignant renal epithelial tumor was diagnosed in one case, and a proteinaceous granulomatous inflammatory effusion was aspirated in the case of urinoma.

## 4. Discussion

Although the diagnosis and management of acute abdomen has been reviewed, there is a lack of information correlating the US findings with the clinical, intraoperative, and histopathologic findings.

There are few studies that provide details on the species, sex, predisposition, duration of signs before presentation, and general condition of patients presenting with acute abdomen syndrome. Our study had more dogs (66%, *n* = 33) than cats (34%, *n* = 17), and male animals were more commonly affected (66%, *n* = 33) than the female animals (34%, *n* = 17). Our results are consistent with a large cohort study which reported that three times more dogs (*n* = 942) were affected than cats (*n* = 309), and that male animals were represented 63.1% (*n* = 790) of the cases [19]. The maximum duration of signs prior to presentation was 10 days in cats and 15 days in dogs [19]. In our study, the mean duration in cats was 4.4 days (ranging 2 h to 10 days) and 2.9 days in dogs (ranging 2 h to 5 days).

The physical examination was used to triage patients with acute abdomen syndrome [6]. In our study, 74% of patients (*n* = 37/50) were classified as mildly to severely impaired at presentation, and two were comatose.

Abdominal palpation is a component in the diagnosis of acute abdominal syndrome [20]. Detection of peritoneal effusion by palpation was confirmed by identification of ascites by US with a sensitivity of 32.4% and a specificity of 93.7%. Abdominal palpation of masses had a sensitivity of 43.7% and a specificity of 94.4%. There was no clinical description of the volume of peritoneal effusion or the size of abdominal masses, thus further studies may be required to show the accuracy of the abdominal palpation. Despite poor performance of abdominal palpation, abdominal palpation may guide clinicians to choose the next diagnostic procedure. Protective abdominal muscle spasms in response to pain may hinder abdominal palpation [21], this may explain the inconclusive palpation findings in 10 animals in the present study.

Survey abdominal radiography did not identify abnormalities in 71% of cases. In patients with peritoneal fluid or peritonitis, loss of intra-abdominal detail makes abdominal radiography less useful for diagnosis of acute abdomen [2]. In a prospective study of human patients with abdominal pain, radiographic diagnosis was correct in 50% of patients [22]. Veterinary studies also describe poor performance of plain abdominal radiography for the diagnosis of abdominal lesions [23,24,25,26]. There is no information about the accuracy of the abdominal radiography in clinical trials of patients with acute abdomen syndrome of different etiologies. Abdominal radiography can guide abdominal US diagnosis and provide information about the advisability of surgical intervention [4].

The present study showed good agreement (86.9%) between US and intraoperative lesions. This is higher than the 64% agreement reported in a study of 100 dogs and cats [16] where diagnosis was confirmed by gross intraoperative observations [16]. In the present study, US was confirmed with cytology (81%) and histopathology (63.3%) [16]. Surgical lesions were not identified by US in 13% (*n* = 12/92) of lesions. The false positive diagnosis by US was reported in 11 lesions (seven primary and four secondary). A higher rate of misdiagnosis (25%) has been reported and gastrointestinal tract ulcerations and perforations were the most commonly misdiagnosed (16). The experience of the examiner and the quality of the machine are important in the interpretation of US findings, as ultrasonography is highly operator and equipment-dependent [27].

Omental tumors were the most commonly misinterpreted lesions including two primary (false positive diagnosis) and three secondary (false negative diagnosis) lesions. The poor sensitivity of abdominal ultrasound for small lesions has been previously reported in human medicine. Macroscopic peritoneal lesions of less than 2 cm are difficult to identify with US and US had a low sensitivity and specificity. Ultrasound cannot replace exploratory laparotomy for the detection of small lesions [15].

Perforation of the GIT was not identified (false negative) by US in two primary and two secondary lesions. Peritoneal effusion is a principal finding in patients with GIT perforation [16]. Ultrasound correctly identified generalized septic peritonitis and ascites. A low sensitivity for US for the identification of GIT perforation has been reported [28,29]. Ultrasound correctly diagnosed spontaneous gastroduodenal perforation in only 1 of 11 dogs [29]. Strong indicators of perforation are free abdominal gas or a combination of local hyperechoic fat and local effusion. In a descriptive retrospective study, GI perforation was listed as a differential diagnosis in 14/19 cases. [28].

One case of GIT foreign body was not detected by US, resulting in a sensitivity of 80%. A prospective study that assessed small intestinal obstruction using specific ultrasonographic criteria correctly diagnosed obstruction in 11 of 13 dogs [30]. Our results suggest that the low number of patients with foreign bodies may have influenced the sensitivity of the US.

Bile duct rupture was not identified (false negative) as primary lesions in two dogs. Although signs of peritonitis, cholangitis, and pericholecystic echogenic reaction were detected by abdominal ultrasound, duct rupture was not listed in the differential diagnosis, suggesting that identifying pericholecystic reaction, and localized or generalized echogenic peritoneal fluid are suspicious for bile duct rupture [31].

Pancreatic neoplasia was documented as the primary lesion intraoperatively in two cases. Abdominal ultrasound indicated the neoplasia preoperatively (*n* = 2). However, the false positive diagnosis was reported in one case; lymph node neoplasia with pancreatitis and general peritonitis were the reported diagnoses in this case. A neoplastic pancreas may appear normal with US or may mimic or be associated with abscessation, pancreatic necrosis, or pancreatitis [32].

In this study, US findings correlated with surgical findings in all primary lesions of the liver, splenic, lymph nodes, and kidney. Although US is regarded as a useful diagnostic modality to recognize abnormalities in these organs, correlation with intraoperative, cytopathologic, and histopathologic findings was not performed [33,34].

Our study is limited by the small sample size, which resulted in some conditions being represented by only one case. A second limitation is that, in several cases, radiographs were obtained prior to US, which may have resulted in preselection of the cases. Also, preoperative knowledge of the US may have influenced the surgical results.

## 5. Conclusions

In conclusion, the study confirmed that abdominal ultrasound is a useful preoperative modality for the diagnosis of acute abdominal disease. Care should be taken with interpretation of US images in animals with gastrointestinal perforation, common bile duct rupture, omental neoplasia, pancreatic neoplasia, and a gastrointestinal foreign body, as these lesions were most commonly misinterpreted with US. Cytology and histopathology confirmed the ultrasound diagnosis in 86.4% of primary and 66.2% of secondary lesions. To determine the correct diagnosis and prognosis, the diagnostic assessment of acute abdomen should combine physical examination (including general condition and abdominal palpation); abdominal imaging (e.g., radiography and ultrasound); and cytopathological analysis.

## Figures and Tables

**Table 1:** Distribution of intraoperative lesions according to anatomical location.

Surgical Diagnosis				
Surgical Diagnosis	Dog	Cat	Lesion Category	
			Primary	Secondary
**Gastrointestinal**				
Intussusception	6	3	9	0
Intestinal neoplasia	2	5	7	0
Foreign body	4	1	5	0
Perforation	1	3	2	2
Ulcer	2	2	0	4
Stomach neoplasia	3	0	3	0
**Peritoneum and peritoneal cavity**				
General peritonitis	8	5	4	9
Ascites	7	2	1	8
Focal peritonitis	6	0	0	6
Omental masses	3	2	2	3
**Pancreas**				
Abscess	5	0	4	1
Neoplasia	2	0	2	0
**Liver**				
Neoplasia	2	2	1	3
Abscess	1	0	1	0
Bile duct rupture	2	0	2	0
**Spleen**				
Neoplasia	4	0	0	4
Abscess	1	0	1	0
**Abdominal LN** ^‡^				
Enlarged LN	4	2	3	3
**Kidney**				
Neoplasia	0	1	1	0
Urinoma	0	1	1	0
Total	63	29	49	43

^‡^ LN: Lymph node.

**Table 2:** Preoperative abdominal ultrasound and intraoperative diagnoses of the primary and secondary lesions based on anatomic location. Sensitivities and specificities (with confidence intervals) of ultrasonography are presented for both primary and secondary lesions identified at surgery. The surgical diagnosis was the “criterion standard”.

Surgical Diagnosis	Ultrasonographic Findings							
Diseases	Primary Lesions				Secondary Lesions			
	Sensitivity (CI *)	Specificity (CI *)	PPV *	NPV *	Sensitivity (CI *)	Specificity (CI *)	PPV *	NPV *
**Gastrointestinal**								
Intussusception	100% CI: 72-100	100% CI: 97–100	100%	100%	-	-	-	-
Intestinal neoplasia	100% CI: 60–100	95% CI: 84–99	77%	100%	-	-	-	-
Foreign body	80% CI: 34–98	97.7% CI: 96–99	80%	98%	-	-	-	-
Perforation	0	0	0	100%	0	0	0	100%
Ulcer	-	-	-	-	100% CI: 34–100	98% CI: 34–98	75%	100%
Stomach neoplasia	100% CI: 72–100	100% CI: 97–100	100%	100%	-	-	-	-
**Peritoneum and peritoneal cavity**								
General Peritonitis								
Ascites	100% CI: 47–100	100% CI: 95–100	100%	100%	89% CI: 60–89	100% CI: 92–100	100%	97%
Focal peritonitis	100% CI: 5.7–100	100% CI: 95–100	100%	100%	100% CI: 68–100	90% CI: 90–97	100%	97%
Omental masses	-	-	-	-	100% CI: 61–100	100% CI: 93–100	100%	100%
	0	95% CI: 86–99	0	100%	0	0	0	100%
**Pancreas**								
Abscess	100% CI: 47–100	97% CI: 84–99	80%	100%	100% CI: 2.5–100	97% CI: 87–99	50%	100%
Neoplasia	100% CI: 56–100	97% CI: 97–98	50%	100%	-	-	-	-
**Liver**								
Neoplasia	100% CI: 47–100	100% CI: 97–100	100%	100%	-	-	-	-
Abscess	100% CI: 57–100	100% CI: 97–100	100%	100%	-	-	-	-
Bile duct rupture	0	0	0	100%	-	-	-	-
**Spleen**								
Neoplasia	-	-	-	-	100% CI: 39–100	94% CI: 82–99	66%	100%
Abscess	100% CI: 57–100	100% CI: 57–100	100%	100%	-	-	-	-
**Abdominal LN** ^‡^								
Enlarged lymph nodes	100% CI: 59–100	100% CI: 96–100	100%	100%	100% CI: 35–100	97% CI: 92–97	75%	100%
**Kidney**								
Neoplasia	100% CI: 57–100	100% CI: 97–100	100%	100%	-	-	-	-
Urinoma	100% CI: 57–100	100% CI: 97–100	100%	100%	-	-	-	-

* CI: Confidence intervals 95%; ^‡^ LN: Lymph node.

**Table 3:** Comparison of ultrasonographic findings with cytopathologic and histopathologic findings. Agreement between the tests is presented as a percentage (%) and number (*n*) of reported ultrasonographic lesions with cytologic or histopathologic confirmation.

Ultrasonography		Preoperative Cytology and Postoperative Histopathology		
Findings	(*n*)	Agreement with US		Findings
		Cytology	Histopathology	
		% (*n*)	% (*n*)	
GIT diseases				
Intussusception	9	100% (9)	100% (9)	Enteritis and lymphadenitis
Intestinal neoplasia	9	55.5% (5)	55.5% (5)	Lymphoma (3) and fibrosarcoma (2)
Ulcer	3	100% (3)	100% (3)	Ulcerative gastritis duo to carcinoma (3)
Stomach neoplasia	3	100% (3)	100% (3)	Carcinoma
Peritoneum and peritoneal cavity				
Peritonitis				
Ascites				
	13	100% (13)	100% (13)	Purulent inflammation (6) and chronic necrotizing peritonitis and omentitis (7)
	9	100% (9)	-	Modified transudate (7) and purulent exudate (2)
Pancreas				
Abscess	7	100% (7)	57% (4)	Purulent inflammation
Neoplasia	1	100% (1)	0	Chronic purulent pancreatitis
Liver				
Neoplasia	1	100% (1)	0	Lymphoma
Nodular hyperplasia	3	100% (3)	100% (3)	Nodular hyperplasia
Abscess	1	100% (1)	0	Purulent hepatitis
Spleen				
Neoplasia	6	0	0	Extra-medullary hematopoiesis (3), regenerating nodules and hyperplasia (3)
				Purulent splenitis
Abscess	1	100% (1)	100% (1)	
Lymph nodes				
Enlarged lymph nodes	6	100% (6)	100% (6)	Purulent lymphadenitis (3), lymphosarcoma (2) and undifferentiated blastoma (1)
Kidney				
Neoplasia	1	100% (1)	100% (1)	Malignant renal epithelial tumor
Urinoma	1	100% (1)	100% (1)	Proteinaceous granulomatous inflammatory effusion
Total	74	86.4% (64)	66.2% (49)	

**Table 4:** Lesions misinterpreted by pre-operative abdominal ultrasound, using surgery as “criterion standard”.

Intraoperative Lesion(s)	Primary Lesions		Secondary Lesions	
	False Negative	False Positive	False Negative	False Positive
Omental mass	-	2	3	-
GIT perforation	2		2	-
Intestinal neoplasia		2		-
Common bile duct rupture	2	-	-	-
Splenic neoplasia	-	-	-	2
Pancreatic abscess	-	1	-	1
Pancreatic tumor	-	1		-
Intestinal foreign body	1	1	-	-
Peritonitis			1	-
GIT ulcer	-	-	1	-
Lymphadenopathy	-	-	-	1
